# Electrochemical Studies for Cation Recognition with Diazo-Coupled Calix[4]arenes

**DOI:** 10.1155/2015/579463

**Published:** 2015-02-17

**Authors:** Bongsu Kim, Tae Hyun Kim

**Affiliations:** Department of Chemistry, Soonchunhyang University, Asan 336-746, Republic of Korea

## Abstract

The electrochemical properties of diazophenylcalix[4]arenes bearing *ortho*-carboxyl group (*o*-CAC) and *ortho*-ester group (*o*-EAC), respectively, in the presence of various metal ions were investigated by voltammetry in CH_3_CN. *o*-CAC and *o*-EAC showed voltammetric changes toward divalent metal ions and no significant changes with monovalent alkali metal ions. However, *o*-CAC preferentially binds with alkaline earth and transition metal ions, whereas no significant changes in voltammetric signals are observed in *o*-EAC with alkaline earth metal ions. *o*-EAC only binds with other transition metal ions. This can be explained on metal ion complexation-induced release of proton from the azophenol to the quinone-hydrazone tautomer followed by internal complexation of the metal ion with aid of nitrogen atoms and *ortho*-carbonyl groups in the diazophenylazocalix[4]arenes.

## 1. Introduction

Macrocyclic receptors have been synthesized and received much attention to be endowed with superior molecular recognition properties used in chemical sensors [1, 2]. Calixarenes have been extensively used as macrocyclic hosts for a wide range of metal ions because they have unique structure of conformational adaptability toward hosting guests along with easy derivatization of the lower rim and upper rim and the possibility of locking a desired conformation (cone, partial cone, 1,2-alternate, and 1,3-alternate) [1, 3, 4]. Many have been utilized as optical sensors to monitor the target by UV/Vis and fluorescence spectroscopic measurements. Among them, azocalixarene bearing azophenol units acting as a chromogenic center are particularly attractive for their interesting aspects of complexation with alkali, alkaline earth, and transition metal ions disclosed by studying the optical behavior of chromophoric units [4–8]. Our group has been also interested in designing selective optical sensors toward metal ions based on azocalixarene derivatives [9, 10]. As one of our efforts, we have developed a simple qualitative analysis protocol to screen alkali, alkaline earth, and transition metal ions using azocalix[4]arene derivatives. To tune up the selectivity toward specific metal ions, we have also tried to perform the pH study of azocalix[4]arene bearing carboxyl group and to change the position or the numbers of substituents, which leads to enhancement of the selectivity for Pb^2+^, Cu^2+^, or Ca^2+^ metal ions in the spectroscopic measurements.

**Scheme 1:**
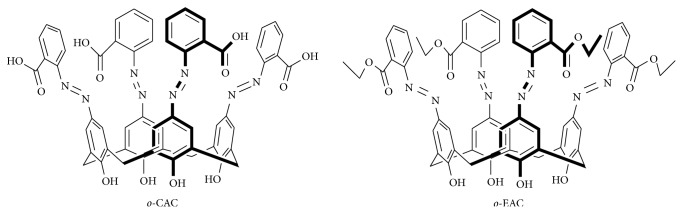
Structures of* ortho*-carboxyl azocalix[4]arene (*o*-CAC) and* ortho*-ester azocalix[4]arene (*o*-EAC).

Azophenols as electroactive groups in azocalixarene system can be studied by also electrochemical measurements [11–13]. Electrochemical sensors are quite interesting and useful because electrochemical changes such as current and voltage by recognition of guest molecule can be directly and immediately reported to electrical signal, which is not necessary to use other transducers. Thus, electrochemical techniques are suitable for the development of convenient, sensitive, selective, and low cost devices that could be utilized for a rapid monitoring, ultimately applicable to hand-held operation. However, only a few examples of electrochemical measurements using azocalix[4]arenes have been reported [10, 14–16]. Previously, our group reported colorimetric discrimination system towards alkali, alkaline earth, and transition metal ions using azophenylcalix[4]arenes bearing* ortho*-carboxyl group (*o*-CAC) and* ortho*-ester group (*o*-EAC) (Scheme 1) [9]. To the best of my knowledge, however, voltammetric study of* o*-CAC and* o*-EAC has not been reported. Present study reports* o*-CAC exhibiting a selective response to alkaline earth and transition metal ions and* o*-EAC to transition metal ions in acetonitrile solution (CH_3_CN) by electrochemical methods. One can discriminate almost all metal ions by simple electroanalytical methods.

## 2. Experimental

### 2.1. Synthesis

Synthesis and identification of* o*-CAC or* o*-EAC were described in the previous papers [9].

#### 2.1.1. 5,11,17,23-*Tetra*[(2-ethyl acetoethoxyphenyl) (azo)phenyl]calix[4]arene (*o*-EAC)

A solution of 2-aminobenzoate (3.10 g, 18.7 mmol), NaNO_2_ (1.46 g, 21.1 mmol), and concentrated HCl (5 mL) in water (10 mL) was added dropwise over a period of about 15 minutes into a solution of calix[4]arene (2.00 g, 4.70 mmol) in THF : pyridine (5 : 2) (25 mL) at 0°C. The solution was stirred for 15 min and then for additional 24 hrs at room temperature. After removal of the solvent* in vacuo*, the resulting solid was dissolved in EtOAc (100 mL) and the organic layer was washed three times with water. The organic layer was dried over anhydrous MgSO_4_ and the solvent was removed* in vacuo* to give a reddish oil. Column chromatography using EtOAc : hexane (2 : 1) provided 2.4 g (45%) of** 1** as a yellow orange solid. Mp: 158–162°C. IR (KBr pellet, cm^−1^): 3220, 1735. ^1^H NMR (200 MHz, CDCl_3_): *δ* 7.43–7.22 (m, 24H, Ar-*H*), 4.24 (d, 4H, Ar-C*H*_*2*_-Ar), 3.97–3.86 (m, 8H, COC*H*_*2*_C*H*_*3*_,* J* = 6.9, 7.3 & 6.7 Hz), 3.42 (d, 4H, Ar-C*H*_*2*_-Ar), 0.82–0.75 (t, 12H, COCH_2_C*H*_*3*_,* J* = 6.9 Hz). ^13^C NMR (DMSO-d_6_): *δ* 168.1, 159.2, 151.5, 145.3, 132.3, 130.8, 129.7, 129.6, 129.0, 124.5, 119.7, 61.5, 32.3, 14.8. FAB MS* m/z* (M^+^): Calcd., 1129.18. Found. 1129.0. Anal. Calcd. for C_64_H_56_N_8_O_12_: C, 68.07; H, 5.00. Found: C, 68.05; H, 5.02.

#### 2.1.2. 5,11,17,23-*Tetra*[(2-benzoic acid)(azo)phenyl]calix[4]arene (*o*-CAC)

A solution of* o*-EAC (0.44 mmol) and NaOH (4.42 mmol) in ethanol (10 mL) and water (5 mL) was refluxed for 12 h and evaporated* in vacuo*. The residue was dissolved in ethyl acetate and the solution was washed twice with 20% HCl and then three times with water. The organic layer was dried over MgSO_4_ and evaporated* in vacuo* to yield 0.35 g (78%) of* o*-CAC as a red solid. Mp: 164–168°C, IR (KBr pellet, cm^−1^): 3220, 1735. ^1^H NMR (200 MHz, DMSO-d_6_): *δ* 10.25 (4H, s, –OH), 7.33–7.02 (m, 24H, Ar-*H*), 4.24 (broad s, 4H, Ar-C*H*_*2*_-Ar), 3.42 (broad s, 4H, Ar-C*H*_*2*_-Ar). ^13^C NMR (DMSO-d_6_): *δ* 30.6, 116.8, 122.4, 130.1, 130.4, 130.9, 134.5, 146.1, 154.5, 155.3, and 169.7. FAB MS* m/z* (M^+^): Calcd. 1016.28. Found. 1016.96. Anal. Calcd. For C_56_H_40_N_8_O_12_: C, 66.14; H, 3.96; N, 11.02; O, 18.88. Found: C, 66.13; H, 3.94; N, 11.03; O, 18.89.

### 2.2. Electrochemical Measurements

Electrochemical measurements were carried out with a Model 660D electrochemical workstation (CH Instruments, Austin, TX, USA). The three-electrode system consists of a glassy carbon working electrode, an Ag/Ag^+^ (in 0.1 M AgNO_3_) reference electrode, and a Pt wire counter electrode. The surface of the working electrode was polished with 0.03 and 0.05 *μ*m alumina (CH Instruments, Austin, TX, USA) and rinsed with deionized water. Residual alumina particles were thoroughly removed by positioning the electrode in an ultrasonic bath for 10 min. Then, the electrode was dried and washed with pure acetonitrile before use. The supporting electrolyte was 0.1 M tetrabutylammonium hexafluorophosphate (TBAPF_6_) in CH_3_CN. The concentration of* o*-CAC or* o*-EAC was 0.1 mM and stock solution of metal perchlorate salts with various concentrations was prepared using acetonitrile. Test solutions were prepared by placing 2 mL of the* o*-CAC or* o*-EAC solution into an electrochemical cell, adding appropriate aliquot of each metal stock solution with a microsyringe. All experiments were carried out in a nitrogen atmosphere at room temperature. All reagents were purchased from Aldrich and used without further purification.

## 3. Results and Discussion

**Figure 1:**
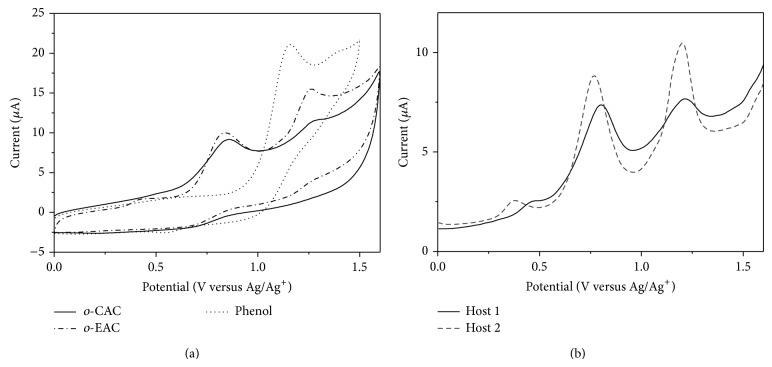
(a) Cyclic voltammograms (CVs) of* o*-CAC,* o*-EAC (1 × 10^−4^ M), and phenol (5 × 10^−4^ M) in 0.1 M TBAPF_6_/CH_3_CN. Scan rate: 50 mV/s. (b) Differential pulse voltammograms (DPVs) of* o*-CAC and* o*-EAC (1 × 10^−4^ M) in 0.1 M TBAPF_6_/CH_3_CN. Pulse amplitude: 50 mV.

The voltammetric behavior of* o*-CAC or* o*-EAC is complicated because the reductions of the azo groups of* o*-CAC or* o*-EAC produce highly proton-sensitive intermediates so that azophenol itself acts as a proton source. The reductions of the azo groups involve two electrons and, however, if a proton source is rich enough to cleave the azo linkage to two –NH_2_ during the electrochemical reduction process, cyclic voltammogram (CV) will give irreversible redox peaks involving four electrons all together [17]. On the other hand, the oxidation of phenol in CH_3_CN shows only one oxidation peak, as shown in Figure 1(a).

Thus, more attention has been paid to the oxidation wave rather than reduction waves of* o*-CAC or* o*-EAC. Electrochemical properties of* o*-CAC or* o*-EAC were also investigated by voltammetry at glassy carbon electrode in 0.1 M TBAPF_6_/CH_3_CN, by taking advantage of the phenol moieties present at the lower rim. Electrochemistry of* o*-CAC or* o*-EAC based on the oxidation of phenols is different from that of phenol (Figure 1(a)). Phenol exhibits simple redox behavior so that an irreversible electron transfer process is observed as one oxidation peak around 1.2 V and calix[4]arene with only phenol functional groups also shows one irreversible oxidation peak [18]. Based on the electrochemistry of phenol,* o*-CAC or* o*-EAC is also expected to be oxidized around 1.2 V. Differently from our prediction,* o*-CAC or* o*-EAC shows two irreversible oxidation waves (Figure 1(a)). This result can be proved by the fact that* o*-CAC or* o*-EAC presents a mixture of the two tautomeric forms, namely, azophenol and quinone-hydrazone, as explained in previous papers by spectroscopic data of* o*-CAC or* o*-EAC [9, 10]. Phenol groups of calixarene with only phenol group are identical because it forms intramolecular hydrogen bonding array in the lower rim of calixarene. But in this case, intramolecular hydrogen bonding array distorts and even breaks by the tautomerization of* o*-CAC or* o*-EAC. Therefore,* o*-CAC or* o*-EAC shows two irreversible peaks at intervals. One peak is due to the ease of protonation of phenol and the other peak is due to the difficulty of oxidation of phenol by intramolecular hydrogen bonding. In order to get a better resolution, differential pulse voltammetry of* o*-CAC or* o*-EAC was also performed in the same condition of cyclic voltammetry. As shown in Figure 1(b), there are two irreversible waves and prewave in the oxidation process of both hosts, which is thought to be due to the tautomerization. Anodic potential of* o*-CAC and* o*-EAC is summarized in Table 1. The potential differences between two peaks (Δ*E*_*p*_ = *E*_*p*2_ − *E*_*p*1_) of* o*-CAC and* o*-EAC are around 400 mV. These values reflect the strength of intramolecular hydrogen bonding. Δ*E*_*p*_s of* o*-CAC from both CV and differential pulse voltammogram (DPV) are slightly smaller than those of* o*-EAC. This might be due to relatively longer distance between two oxygens of* o*-CAC than that of* o*-EAC caused by the relative degree of steric hindrance between* o*-carboxyl groups and* o*-ester groups.

**Figure 2:**
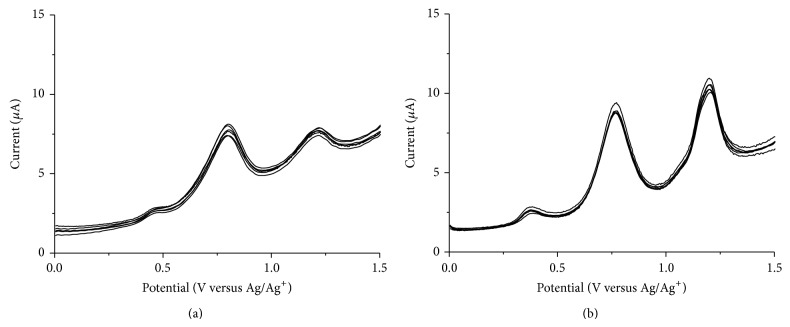
DPVs of (a)* o*-CAC and (b)* o*-EAC (1 × 10^−4^ M) in the absence and presence of alkali metal ions (1 × 10^−4^ M).

**Figure 3:**
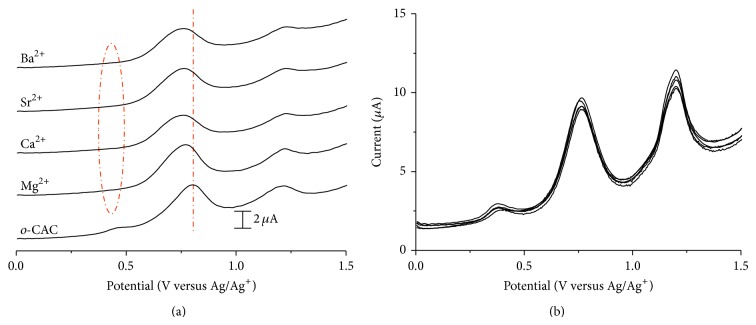
DPVs of (a)* o*-CAC and (b)* o*-EAC (1 × 10^−4^ M) in the absence and presence of alkaline earth metal ions (1 × 10^−4^ M).

**Figure 4:**
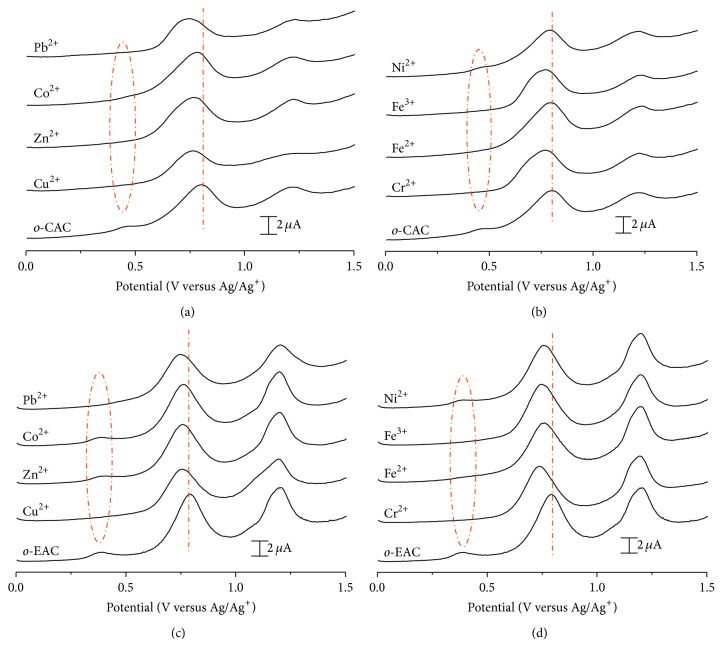
DPVs of ((a), (b))* o*-CAC (1 × 10^−4^ M) and ((c), (d))* o*-EAC (1 × 10^−4^ M) in the absence and presence of transition metal ions (1 × 10^−4^ M). Pulse amplitude: 50 mV, pulse width: 0.05 sec, sample width: 0.0167 sec, and pulse period: 0.2 sec.

**Figure 5:**
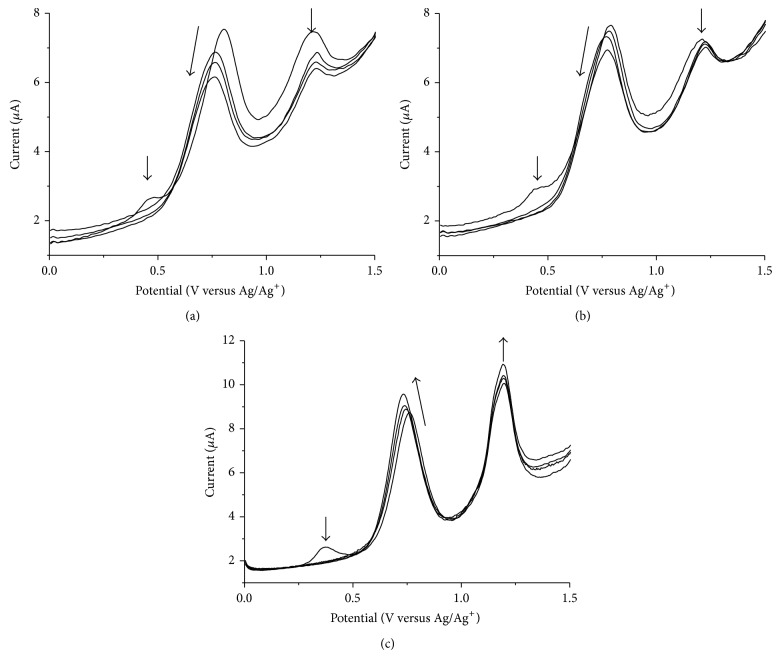
DPVs of* o*-CAC (1 × 10^−4^ M) upon the addition of increasing amount of (a) Sr^2+^ and (b) Zn^2+^, and (c) DPVs of* o*-EAC upon the addition of increasing amount of Cr^2+^. [M^2+^] = 0.5, 1.0, and 1.5 × 10^−4^ M. Pulse amplitude: 50 mV, pulse width: 0.05 sec, sample width: 0.0167 sec, and pulse period: 0.2 sec.

The complexation behavior of* o*-CAC and* o*-EAC was also investigated in the presence of alkali, alkaline earth, and transition metal ions in CH_3_CN by comparing the voltammetric behaviors of their phenols in differential pulse voltammetry with 0.1 M TBAPF_6_ as the supporting electrolyte. A constant volume (10 *μ*L) of each cation stock solution was injected successively into the electrochemical solution to make 0.5–1.5 equivalent of cation in the solution. DPVs were recorded after adding stoichiometric equivalent of metal ions successively to the respective electrochemical solution. DPVs of 0.1 mM* o*-CAC and* o*-EAC in the presence of one equivalent of alkali and alkaline earth metal ions are shown in Figures 2–5.

Successive addition of an alkali metal ion to* o*-CAC or* o*-EAC, which is incapable of encapsulation, caused no significant change in the peak current or potential in accordance with previous spectroscopic data [9].  Figure 2 shows typical DPVs of* o*-CAC and* o*-EAC with the addition of one equivalent of alkali metal ions.

Very similar results are observed with* o*-EAC–alkaline earth metal complexation (Figure 3(b)). On the other hand,* o*-CAC in the presence of alkaline earth metal ions shows changes in peak currents and peak potentials (Figure 3(a)). Preoxidation wave around 0.5 V disappears and first oxidation peak shifts at more negative potential (Figure 3(a)). The presence of transition metal ions also alters both the oxidation peak potentials and currents of* o*-CAC or* o*-EAC, as in the voltammetric behavior of* o*-CAC–alkaline earth cation complexation (Figure 4). This result indicates that there is a subtle balance between metal complexation-induced release of proton from the azophenol to the quinone-hydrazone tautomer, and the* ortho*-carboxyl groups of* o*-CAC or* ortho*-ester groups of* o*-EAC can stabilize the quinone-hydrazone form in azocalix[4]arene after adding alkaline earth or transition metal ions.

The peak potential differences between* o*-CAC or* o*-EAC and* o*-CAC– or* o*-EAC–metal ion complexes are summarized in Table 2. The presence of alkaline earth and transition metal ions affects the oxidation peak potentials of* o*-CAC, and transition metal ions influence the oxidation behavior of* o*-EAC, whereas all alkali metal ions lead to no noticeable change in the voltammetric behavior of* o*-CAC and* o*-EAC. This result is in good agreement with previous spectroscopic experiments [9].

**Scheme 2:**
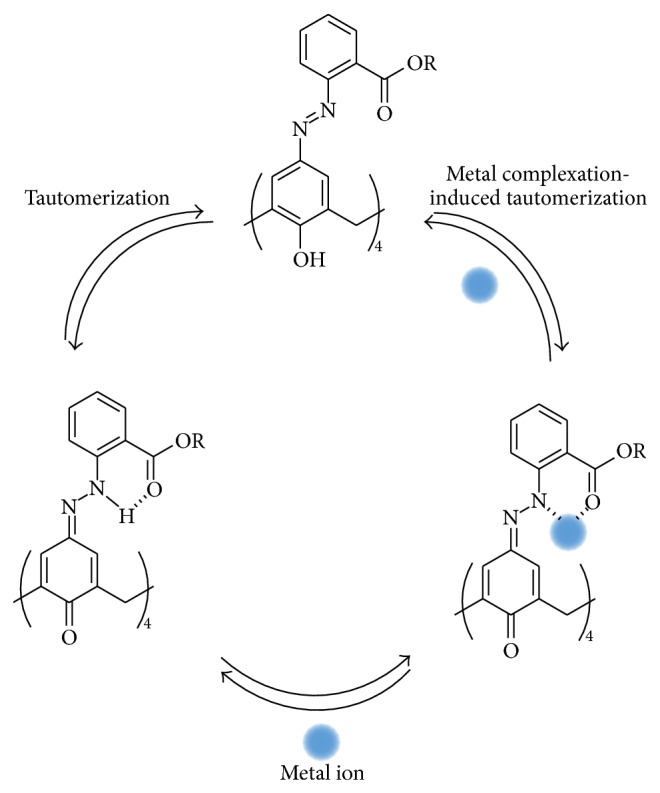
Possible complexation mechanism of* o*-CAC and* o*-EAC with metal ion.

In order to confirm this electrochemical recognition phenomenon, the electroactivities of* o*-CAC or* o*-EAC were measured in the presence of increasing substoichiometric amount of Sr^2+^, Zn^2+^, or Cr^2+^ cations, as representative models of alkaline earth or transition metal ions. The behavior observed in the oxidation process is shown in Figure 5. With a function of concentration of metal ion, preoxidation waves around 0.5 V of both* o*-CAC and* o*-EAC disappear and first oxidation peaks shift at more negative potential. While second oxidation peak of both* o*-CAC and* o*-EAC decreases, first oxidation peak of* o*-EAC, however, increases and* o*-CAC decreases. This implies the metal complexation-induced release of protons from the azophenol to the quinone-hydrazone tautomer, but the tautomerization to the quinone-hydrazone becomes somewhat faster in* o*-EAC and slower in* o*-CAC, as the concentration of metal ions increases. From this, the possible complexation mechanism based on the 1 : 1 complexation is proposed in Scheme 2.

## 4. Conclusion

The electrochemical behaviors of azocalix[4]arene derivatives containing* ortho*-carboxyl or diethyl ester groups have been investigated in the absence and the presence of alkali, alkaline earth, and transition metal ions by electrochemical measurements.* o*-CAC with the* ortho*-carboxyl groups preferentially binds with alkaline earth and transition metal ions over alkali metal ions, whereas* o*-EAC with the* ortho*-diethyl ester groups shows selective complexation properties toward transition metal ions over alkali and alkaline earth metal ions. The complexation of metal ions gives rise to negative shifts of first oxidation peaks in the electrochemistry of the azocalix[4]arenes. This may be attributed to the metal complexation-induced release of protons from the azophenol to the quinone-hydrazone tautomer. With this system, one can screen metal ions to alkali, alkaline earth, and transition metal ions using simple electrochemical methods.

## Figures and Tables

**Table 1:** Anodic peak potentials of *o*-CAC and *o*-EAC.

	*o*-CAC		*o*-EAC	
	CV	DPV	CV	DPV
*E*_pre_	—	460	424	376
*E*_*p*1_	861	804	840	776
*E*_*p*2_	1277	1212	1262	1204
Δ*E*_*p*_ (= *E*_*p*2_ − *E*_*p*1_)	416	408	422	428

**Table 2:** Anodic peak potential differences between *o*-CAC or *o*-EAC and *o*-CAC– or *o*-EAC–metal ion complexes in DPVs.

mV		Li^+^	Na^+^	K^+^	Rb^+^	Cs^+^	Mg^2+^	Ca^2+^	Sr^2+^	Ba^2+^	Cu^2+^	Zn^2+^	Co^2+^	Pb^2+^	Cr^2+^	Fe^2+^	Fe^3+^	Ni^2+^
*o*-CAC	Δ*E*_*p*1_	−4	0	−8	0	−4	−32	−44	−40	−44	−40	−36	−20	−56	−32	−8	−32	−12
	Δ*E*_*p*2_	12	4	4	4	8	12	16	16	16	4	12	12	20	12	24	24	8

*o*-EAC	Δ*E*_*p*1_	−4	−8	−12	−8	−4	−20	−12	−12	−12	−16	−20	−12	−28	−36	−16	−28	−20
	Δ*E*_*p*2_	−4	0	−4	−4	−4	−4	0	−4	−4	−8	−4	−4	0	−8	0	−4	−4
